# Development and Application of a Target Capture Sequencing SNP-Genotyping Platform in Rice

**DOI:** 10.3390/genes13050794

**Published:** 2022-04-28

**Authors:** Chaewon Lee, Kyeong-Seong Cheon, Yunji Shin, Hyoja Oh, Young-Min Jeong, Hoon Jang, Yong-Chan Park, Kyung-Yun Kim, Hang-Chul Cho, Yong-Jae Won, Jeongho Baek, Young-Soon Cha, Song-Lim Kim, Kyung-Hwan Kim, Hyeonso Ji

**Affiliations:** 1 Department of Agricultural Biotechnology, National Institute of Agricultural Sciences, Rural Development Administration (RDA), Jeonju 54874, Korea; wowlek44@korea.kr (C.L.); yunji.shin8@gmail.com (Y.S.); hja-oh@hanmail.net (H.O.); firstleon@korea.kr (J.B.); yscha63@korea.kr (Y.-S.C.); greenksl@korea.kr (S.-L.K.); biopiakim@korea.kr (K.-H.K.); 2 Department of Crop Science and Biotechnology, Chonbuk National University, Jeonju 54896, Korea; 3 Division of Forest Tree Improvement and Biotechnology, Department of Forest Bioresources, National Institute of Forest Science, Suwon 16631, Korea; kscheon16@korea.kr; 4 Genecell Biotech Inc., Wanju, 55322, Korea; 5 Seed Industry Promotion Center, Korea Agriculture Technology Promotion Agency (KOAT), Gimje 54324, Korea; ymjeong@koat.or.kr; 6 CELEMICS, Seoul 08506, Korea; hoon.jang@celemics.com (H.J.); ycpark@celemics.com (Y.-C.P.); 7 INSILICOGEN, Yongin 16954, Korea; kykim@insilicogen.com (K.-Y.K.); hccho@insilicogen.com (H.-C.C.); 8 Cheorwon Branch, National Institute of Crop Science, Rural Development Administration (RDA), Cheorwon 24010, Korea; yjwon@korea.kr

**Keywords:** genotyping, preharvest sprouting, QTL, rice, SNP, target capture sequencing

## Abstract

The development of efficient, robust, and high-throughput SNP genotyping platforms is pivotal for crop genetics and breeding. Recently, SNP genotyping platforms based on target capture sequencing, which is very flexible in terms of the number of SNP markers, have been developed for maize, cassava, and fava bean. We aimed to develop a target capture sequencing SNP genotyping platform for rice. A target capture sequencing panel containing 2565 SNPs, including 1225 SNPs informative for *japonica* and 1339 SNPs informative for *indica*, was developed. This platform was used in diversity analysis of 50 rice varieties. Of the 2565 SNP markers, 2341 (91.3%) produced useful polymorphic genotype data, enabling the production of a phylogenetic tree of the 50 varieties. The mean number of markers polymorphic between any two varieties was 854. The platform was used for QTL mapping of preharvest sprouting (PHS) resistance in an F_8_ recombinant inbred line population derived from the cross Odae × Joun. A genetic map comprising 475 markers was constructed, and two QTLs for PHS resistance were identified on chromosomes 4 and 11. This system is a powerful tool for rice genetics and breeding and will facilitate QTL studies and gene mapping, germplasm diversity analysis, and marker-assisted selection.

## 1. Introduction

Rice (*Oryza sativa*) is an essential component of the diet of over 3.5 billion people [1]. Worldwide, nearly 504.7 million metric tons of milled rice were produced from ~164.2 million hectares of paddy fields in 2020 (http://www.fao.org/faostat, accessed 18 January 2022). The predicted increase in the global population to ~10 billion by 2050 demands urgent improvements in rice productivity. At the same time, rice breeding programs must develop varieties that require less fertilizer and are resistant to the intensifying biotic and abiotic stresses resulting from global climate change [1].

Single-nucleotide polymorphism (SNP) markers are now the most commonly used DNA marker type for assisting crop genetics and breeding. SNPs possess several characteristics useful in DNA markers, being abundant, codominant, and evenly distributed across plant genomes. Mapping of genes or quantitative trait loci (QTLs), genome-wide association studies (GWAS), germplasm diversity analysis, marker-assisted selection, and genomic selection studies are nowadays most frequently performed using SNP genotyping. Therefore, the development of efficient, robust, and high-throughput SNP genotyping platforms is pivotal for crop genetics and breeding.

Arrays and next-generation sequencing (NGS) are two major high-throughput SNP genotyping platforms [2]. Various high-throughput arrays for genotyping SNPs have been developed for rice. They can be classified into low-, medium-, and high-density arrays according to the number of SNP markers included. Existing low- and medium-density arrays include an Illumina GoldenGate array consisting of 1536 SNPs [3]; a suite of Illumina BeadXpress arrays including 384 SNPs [4]; an Illumina GoldenGate array for diversity and genetic analysis of Japanese temperate *japonica* rice varieties consisting of 768 SNPs [5]; two Illumina Infinium-based 6 K arrays, RiceSNP6K [6] and C6AIR [7]; and the C7AIR SNP array containing 7098 markers, which is an enhancement of the C6AIR array [8]. These arrays have all been used successfully for studies including QTL mapping, diversity analysis, and marker-assisted backcross breeding (MABB). High-density arrays include the Affymetrix array, which consists of 44,100 SNPs [9]; two 50 K arrays, RiceSNP50 [2] and OsSNPnks [10]; and the 700 K High-Density Rice Array (HDRA700K) [11]. These arrays have mainly been used for GWAS analyses.

Genotyping by sequencing (GBS) is an NGS-based SNP genotyping platform that has been widely used for genetic studies, including GWAS, gene and QTL mapping, and diversity analysis, because it is highly efficient and cost-effective. GBS-based approaches require considerable bioinformatics support, however [12]. Another NGS-based SNP genotyping platform is Targeted Amplicon Sequencing (TAS), in which several hundred to a few thousand target SNP markers are genotyped by sequencing multiplex PCR-based amplicons [13]. The 1000-SNP (1 K) Rice Custom Amplicon assay, or 1k-RiCA, was developed for genetic and breeding purposes using highly informative SNPs from *indica* rice breeding pools [13]. Other SNP genotyping assays using target capture sequencing technology have been developed for crop plants. With this technology, DNA fragments including a 100-200 bp region of each SNP marker were captured by two to four probes and sequenced using next-generation sequencing. This technology is both very flexible, as it is available for both small and large numbers of markers, and easy to improve by adding new SNPs to the probe panels. It has been used to develop genotyping systems with 1 K, 5 K, 10 K, 20 K, and 251 K SNP marker panels in maize (*Zea mays*) [14,15]. In addition, a 130 K SNP genotyping platform using this technology was developed in faba bean (*Vicia faba*) [16], and a 27,469 SNP marker probe panel was used successfully for a QTL analysis of starch viscosity traits in cassava (*Manihot esculenta* Crantz) [17]. Moreover, a core Kompetitive Allele-Specific PCR (KASP) array that included 467 informative markers was used successfully to assess germplasm and genetic diversity and evaluate populations in rice [18]. 

We previously developed 1225 KASP markers based on SNPs detected by resequencing 13 Korean temperate *japonica* rice varieties [19,20,21]. These markers have enabled QTL mapping of several important traits, including bakanae disease resistance [22], preharvest sprouting (PHS) resistance [23], seed size [24], and spikelet sterility [25], as well as for MABB programs [26,27,28] in temperate *japonica* rice varieties. These markers, however, were not useful for analyzing variation in *indica* rice due to their low polymorphism in *indica* varieties. This motivated us to develop an SNP genotyping platform suited to both *japonica* and *indica* varieties.

In this study, we aimed to develop a novel SNP genotyping platform for rice that is applicable to both *japonica* and *indica*, based on target capture sequencing technology. We developed a target capture sequencing panel including 2565 SNPs: the SNPs used to develop the 1225 KASP markers for temperate *japonica* varieties [19,20,21]; the 1339 SNPs included in the 1k-RiCA [13] and the core KASP array [18], which are informative for *indica* varieties; and an SNP located in the *semi dwarf 1* (*sd1*) gene [29]. This system was used in a diversity analysis of 50 rice varieties and for QTL mapping of PHS resistance. This platform is suited to use across a wide range of rice varieties because it includes informative SNPs for both *japonica* and *indica* varieties. It provides an important contribution to rice genetics and breeding that will facilitate QTL and gene mapping, analyses of germplasm diversity, and marker-assisted selection. 

## 2. Materials and Methods

### 2.1. Plant Materials

Seeds of the 50 rice varieties listed in Supplementary Table S1 and from 162 F8 recombinant inbred lines (RILs) derived from the cross Odae × Joun were sown in seedling trays. After 1 month, 20 seedlings per variety and RIL were transplanted to the experimental field of the National Institute of Agricultural Sciences (NIAS) of the Rural Development Administration (RDA, Jeonju, Korea). Leaf samples for DNA extraction were collected from healthy plants about 1 month after transplanting. DNA was extracted using the DNeasy Plant Mini Kit (QIAGEN, Hilden, Germany).

### 2.2. Target Capture Panel Design and Next-generation Sequencing

In total, 2565 SNPs were included in the target capture panel design. These included the SNPs used to develop 1225 KASP markers for temperate *japonica* varieties [19,20,21]. To ensure SNPs informative for *indica* varieties were also present, the 895 SNPs included in the 1k-RiCA [13] and the 467 SNPs from the core KASP array [18] were included in the target capture panel design. Once duplicated SNPs from the 1k-RiCA and the core KASP array had been removed, 1339 *indica* SNPs were included in the panel. Finally, an SNP from *sd1* [29] was included. The target capture panel and reagent kits were manufactured based on liquid-phase hybridization technology by Celemics, Inc. (Seoul, Korea). For each selected SNP, surrounding 60 bp regions were defined as targets. Capture probes were designed to complementarily bind to the corresponding target region. Probes for the same target partially overlapped with each other so that each SNP could be captured by at least two different probes. The rice capture panel was manufactured by chemically synthesizing the designed probes. Each probe contained a biotin molecule and therefore could be recovered using streptavidin beads after binding to the target. 

Genomic DNA was cut into approximately 250 bp fragments using a Bioruptor Pico Sonication System (Diagenode, Liege, Belgium) and processed for Illumina sequencing by the following steps: end-repair, dA-tailing, adapter ligation, and pre-PCR for indexed NGS library. The prepared gDNA library and capture probes were hybridized to capture target regions using the Celemics target enrichment kit (Celemics, Seoul, Korea). Target-captured libraries were amplified by post-PCR to enrich the amount of sample and sequenced on an Illumina NextSeq550 instrument (Illumina, San Diego, CA, USA) using the read layout 2 × 150 bp. The raw sequencing data were analyzed using the CLC Genomics Workbench program version 21 (QIAGEN, Hilden, Germany).

### 2.3. Variety Diversity Analysis and QTL Mapping

The polymorphism information content (*PIC*) value of markers was calculated using the following equation, which is applicable to inbred varieties [30]:

$$PIC=1-\overset{n}{\underset{i=1}{\sum }}Pi^{2}$$

The population structure of the 50 varieties was determined using the STRUCTURE (version 2.3.4) [31,32] program and by varying the number of clusters (K) between one and eight, with ten replications. A model following admixture and correlated allele frequency with a 5000 burn-in length and a run length of 50,000 was used for model-based structure analysis. The output of the STRUCTURE analysis was collected using STRUCTURE harvester [33], and the most probable K value was determined based on the LnP(D) and Evanno’s ΔK [34]. Phylogenetic analysis of the 50 varieties was performed using the MEGA X program [35]. The evolutionary history was inferred during phylogenetic analysis using the Neighbor-Joining method [36], and the evolutionary distances were computed using the Maximum Composite Likelihood method [37].

For a QTL analysis of PHS resistance, 162 RILs derived from an Odae × Joun cross were grown in a greenhouse with maximum/minimum temperatures of 32 °C/22 °C and light/dark periods of 14 h/10 h. Seeds of both parental varieties and 162 RILs were sown in 50-hole seedling raising trays. Seedlings were transferred to pots (150 mm diameter; 125 mm height) approximately 4 weeks after sowing; a total of six seedlings per line were transferred to two pots (three plants per pot). During plant growth, the main culm was retained on each plant, but all tillers were removed. This ensured PHS was measured in panicles from main culms of plants, thus eliminating variation in PHS caused by different tiller positions. PHS rates were evaluated according to the method of Cheon et al. [23]. The heading panicles were tagged by wrapping their culms with colored tape, and heading dates were recorded. Panicles were harvested from six plants per line (one panicle from each plant) 38 days after heading, when the cumulative daily mean temperature during seed ripening reached 1000 °C. The panicles were placed on paper towels on stainless steel trays (465 × 365 × 35 mm), and tap water was added until the panicles were slightly immersed. Paper towels were spread over the panicles, and then the tray was covered with plastic wrap to prevent water loss through evaporation and placed in an incubator at 25 °C. After 7 days, the panicles were removed from the tray, and the numbers of germinated and nongerminated seeds per panicle were counted. Any seed with a shoot that had emerged from a break in the husk was considered to have germinated. The PHS rate (%) was calculated as (germinated seeds/total filled seeds) × 100. The average PHS rate for each RIL was calculated from four panicles per line, removing maximum and minimum PHS rate panicles, and used for QTL analysis. The QTL IciMapping 4.1 program [38] was used to construct a genetic map and perform QTL analysis. The Kosambi function was used for mapping. The LOD threshold was calculated using 1000× permutations.

## 3. Results

### 3.1. Target Capture Panel Design

Our target capture panel contained, in total, 2565 SNP markers: 1225 SNPs used to develop KASP markers for temperate japonica rice varieties; 1339 SNPs from the 1k-RiCA [13] and the core KASP array [18], which cover indica varieties; and an SNP from sd1. The positions of all the SNP markers in the rice reference genome are listed in Supplementary Table S2.

### 3.2. Diversity Analysis of Rice Varieties Using the Target Capture Panel

**Figure 1:**
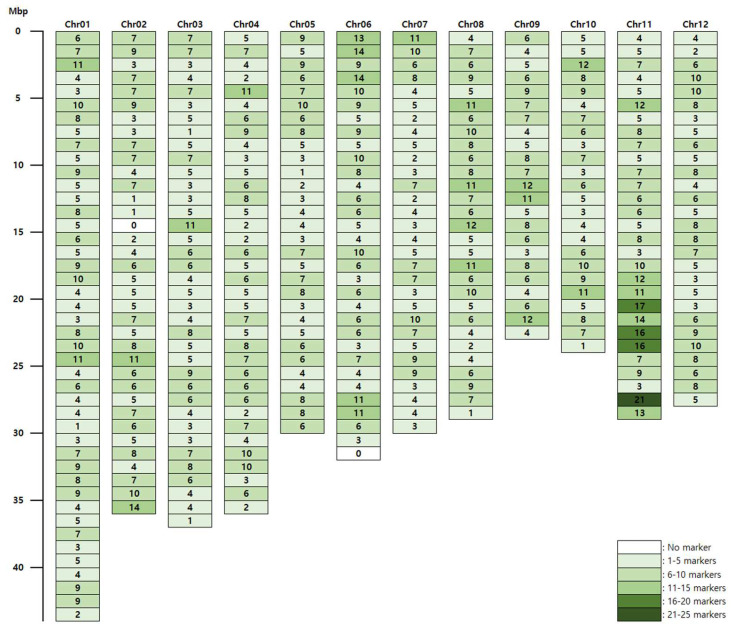
Distribution of SNP markers included in the target capture panel across the rice reference genome. Columns represent chromosomes, and rows represent physical positions (1 Mbp intervals). The numbers of SNP markers per Mbp are shown in each row; cell colors differ according to SNP marker density, as indicated in the legend in the lower right-hand corner of the figure.

**Figure 2:**
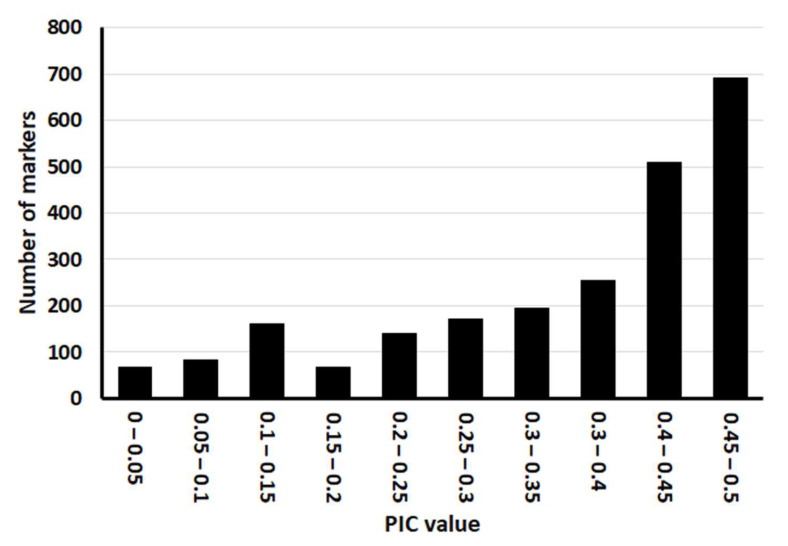
Polymorphism information content (PIC) values of SNP markers present on the target capture panel.

We analyzed 50 rice varieties (29 japonica and 21 indica varieties; Supplementary Table S1) using our novel target capture panel. The genotype data for the 50 rice varieties and the PIC value of each marker are shown in Supplementary Table S3. Of the 2565 SNP markers present in the target capture panel, 2341 (91.3%) produced useful polymorphic genotype data; over the 50 varieties tested, 2334 of the markers produced no missing data, 6 markers missed one datapoint, and 1 marker missed two datapoints. We therefore concluded that the target capture panel contained 2341 informative SNP markers. The distribution of these markers across the rice reference genome is shown in Figure 1. Overall, the markers were distributed relatively evenly, with an average density of 6.3 markers/Mbp (Supplementary Table S4). The PIC value ranged from 0.039 to 0.5 (mean: 0.356). The distribution of PIC values is shown in Figure 2; there were more markers with higher PIC values than markers with lower ones.

The number of markers polymorphic between two varieties was calculated for all variety pairs (Table 1). It ranged from 159 to 1368 (mean: 854) across all varieties, although within the japonica group, the range was 159–916 (mean: 623), and within the indica group, the range was 162–861 (mean: 491). When japonica and indica varieties were compared, the number of markers polymorphic between two varieties ranged from 603 to 1368 (mean: 1177). These results indicate that the markers on the target capture panel could acquire sufficient genotypic data for gene or QTL mapping or for MABB from almost all combinations of varieties.

**Figure 3:**
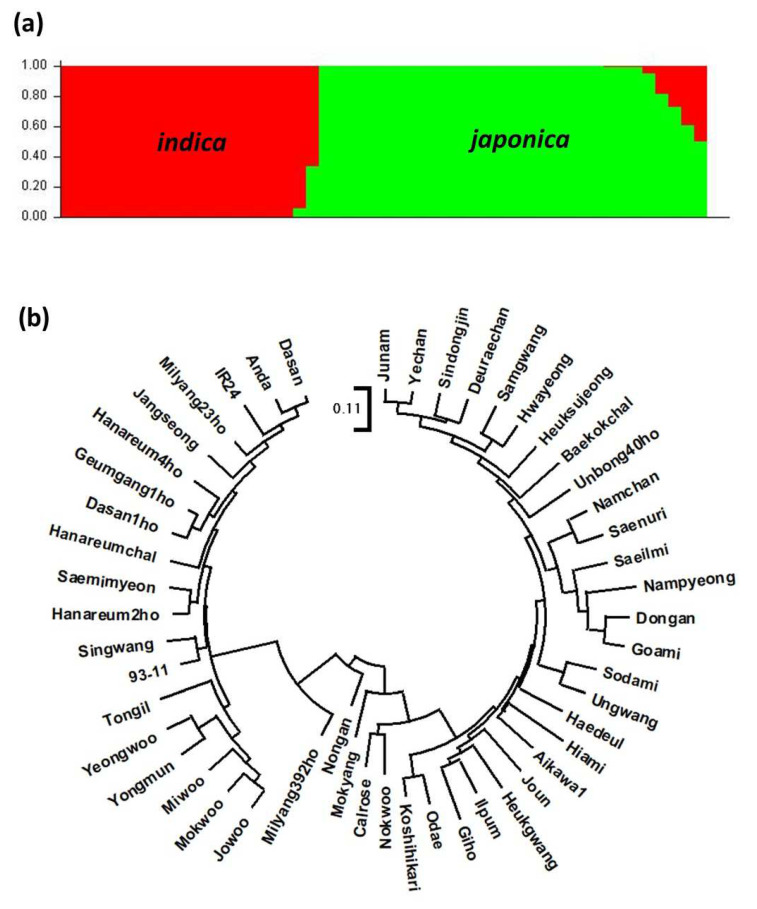
Population structure and phylogeny analysis of the 50 rice varieties used in this study. (**a**) Assignment of the rice varieties to two populations (japonica and indica) using the STRUCTURE 2.3.4 program. (**b**) Phylogenetic tree of the rice varieties. Their evolutionary history was inferred using the Neighbor-Joining method. The optimal tree with the sum of branch length = 5.76867357 is shown. The tree is drawn to scale, with branch lengths in the same units as those of the evolutionary distances used to infer the phylogenetic tree. The evolutionary distances were computed using the Maximum Composite Likelihood method and are in the units of the number of base substitutions per site.

We used the STRUCTURE 2.3.4 program to analyze the population structure of the 50 rice varieties [31,32]. Two distinct populations were identified by Evanno’s ΔK values (i.e., K = 2; Supplementary Figure S1), which corresponded to the japonica and indica subspecies (Figure 3a). A phylogenetic tree of the 50 rice varieties was constructed using the MEGA X program (Figure 3b). Most varieties of japonica and indica were clearly separated, although three varieties (Mokyang, Nongan, and Milyang392ho) were of the intermediate type.

### 3.3. QTL Analysis of Preharvest Sprouting Resistance Using the Target Capture Panel

**Figure 4:**
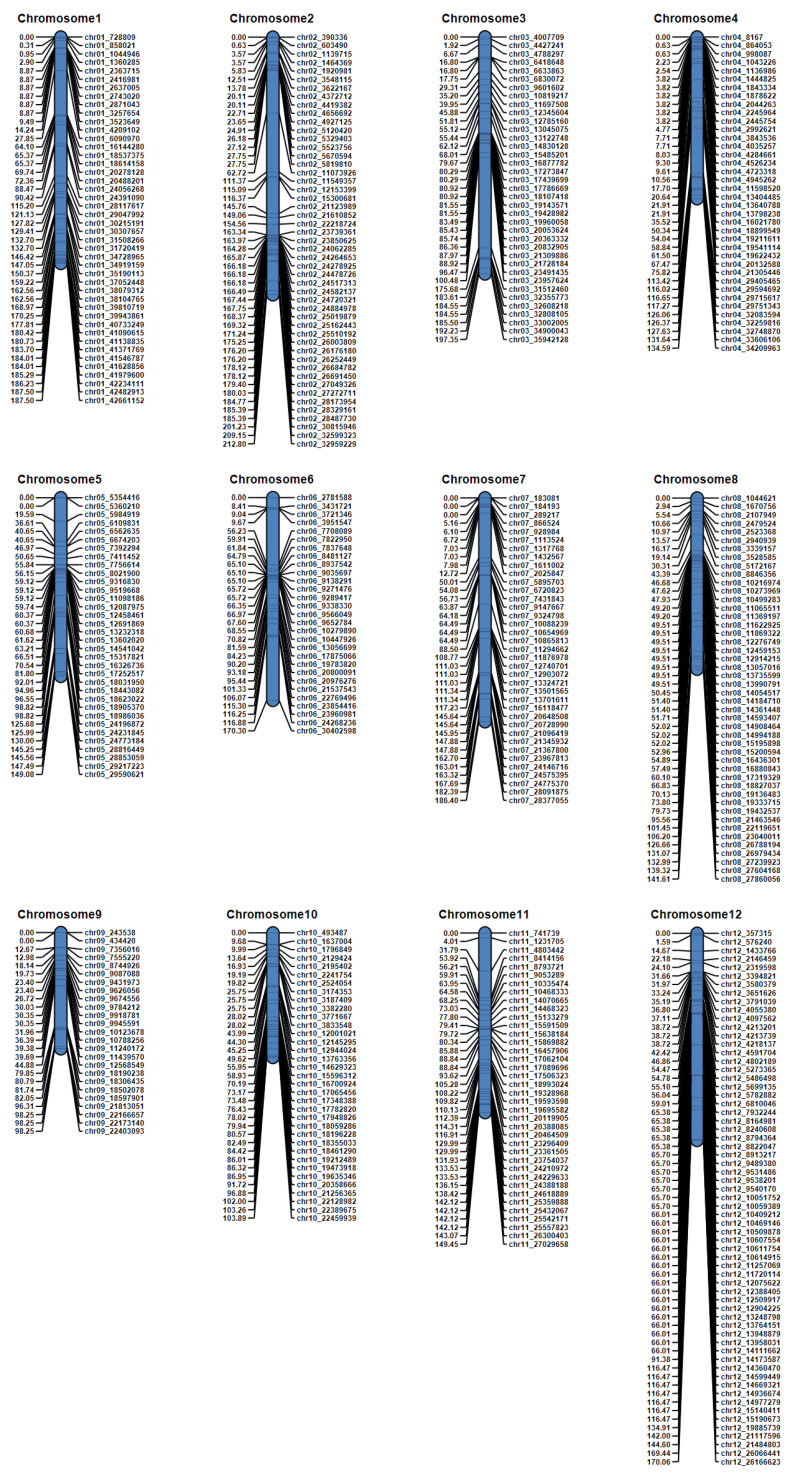
Construction of a genetic map using genotype data from 162 F_8_ recombinant inbred lines (RILs) derived from an Odae × Joun cross obtained from the target capture panel. Chromosomes are numbered at the top. Marker names are indicated on the right-hand side of each chromosome, and the genetic distance of each marker from the first marker at the top of the chromosome is shown on the left-hand side. Genetic distances, measured in cM, were calculated using the Kosambi function.

To test the usefulness of the target capture panel for QTL mapping, we performed a QTL analysis of PHS resistance using 162 RILs derived from an Odae × Joun cross. Both parents are Korean temperate japonica rice varieties; Odae is moderately resistant to PHS, and Joun is resistant to PHS. We genotyped the RIL population using the target capture panel and found 475 SNP markers produced polymorphic genotype data, enabling construction of a genetic map (Figure 4). The total length of this genetic map was 1901.3 cM, with a mean interval between markers of 4.11 cM.

**Figure 5:**
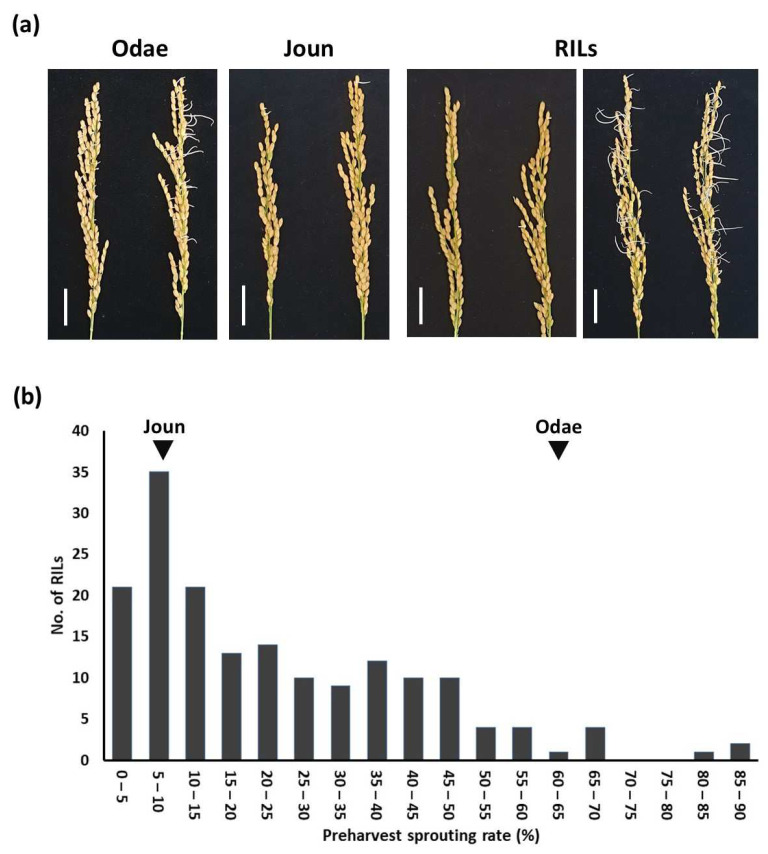
Phenotypic variation in preharvest sprouting (PHS) in the rice varieties Odae and Joun and a recombinant inbred line (RIL) population derived from an Odae × Joun cross. (**a**) PHS phenotypes of the parental varieties and representative RILs. Scale bar: 20 mm. (**b**) Distribution of PHS rates across 162 RILs and their parental lines. Inverted triangles indicate the parental varieties.

We measured PHS rates in the RILs and the parental varieties, Odae and Joun. PHS rates differed, both between the parental varieties and within the RIL population (Figure 5a). The distribution of PHS rates across the 162 F_8_ RILs is shown in Figure 5b.

**Figure 6:**
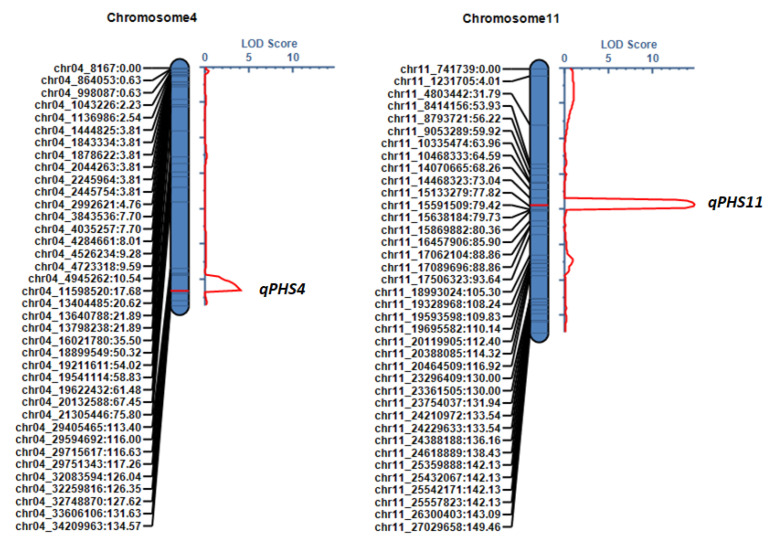
Positions of two QTLs affecting preharvest sprouting (PHS) rate in the genetic map of 162 RILs derived from the cross Odae × Joun. The names of each marker and their genetic distances from the first marker at the top of each chromosome are shown on the left-hand side. The LOD score (red line) is shown on the right-hand side.

A QTL analysis of the PHS rate was performed using the genetic map and phenotypic data obtained from the 162 RILs derived from the Odae × Joun cross. This analysis identified two QTLs: qPHS4, located 126 cM from the top of chromosome 4, and qPHS11, located 77 cM from the top of chromosome 11. The logarithm-of-the-odds (LOD) scores of these QTLs were 4.06 and 14.74, respectively (Table 2; Figure 6). The LOD threshold was determined to be 3.704 through 1000× permutations. The additive effects of these two QTLs were −4.84 and −10.13, respectively, with 6.48% and 28.66% of phenotypic variation, respectively, being explained by QTL effects (PVE); Joun alleles decreased the PHS rate at both QTLs. The QTL intervals (95% probability) were 121.5–126.5 cM for qPHS4 and 74.5–78.5 cM for qPHS11 (Table 2). The qPHS4 region was flanked by the markers chr04_29751343 and chr04_32748870 (29.751–32.749 Mbp) on chromosome 4, whereas the qPHS11 region was flanked by chr11_14468323 and chr11_15591509 (14.468–15.592 Mbp) on chromosome 11.

## 4. Discussion

We successfully developed a target capture sequencing platform for genotyping SNPs in rice. This platform produced useful polymorphic genotype data for 2341 SNP markers across 50 different rice varieties (29 *japonica* and 21 *indica*), providing a mean of 854 for every combination of two varieties tested. Most previous QTL or gene mapping studies in rice have used traditional markers such as SSR and RFLP and involved 100–300 polymorphic markers. Our novel platform thus contained a sufficient number of polymorphic markers for QTL and gene mapping studies, MABB, and germplasm diversity analysis. In addition, the mean PIC value of markers in our platform was fairly high (0.356), while all PIC values were in the range 0–0.5 (Figure 2). The majority of markers had high PIC values (Figure 2), which indicated most of the markers present in this platform were highly polymorphic between rice varieties and therefore provided informative genotypic data. In addition, this platform produced very few missing datapoints; of a total of 117,050 datapoints obtained from 50 varieties using 2341 markers, only 8 (0.007%) were missing. These results indicate that the target capture sequencing SNP genotyping platform developed in this study offers a very powerful tool for various types of genetic studies, including QTL and gene mapping analyses and the development of breeding programs.

A target capture sequencing SNP genotyping platform is highly flexible because it is very easy to add liquid probes to an existing panel [14,15,16]. The mean number of markers revealing polymorphisms in *indica* varieties was lower than for *japonica* varieties (491 vs. 623) in the rice platform described here. Most of the *indica* varieties used in this study were Korean Tongil-type varieties, which have low genetic diversity as there is far less breeding of Tongil-type rice than of *japonica* rice in Korea. The requirement to develop new lines of Tongil-type rice is increasing in Korea, however, as these varieties are suited to diverse uses, including livestock feed and raw material for processed foods such as rice noodles and cookies. It is therefore important to ensure a sufficient number of suitable markers is available to enable breeding of such *indica* rice varieties. This will involve the development of markers capable of detecting polymorphisms within Tongil-type varieties by using genome sequence analysis to identify highly polymorphic SNPs within representative Korean Tongil-type varieties.

PHS occurs when seeds break dormancy under warm and humid circumstances prior to harvest. It is observed in many cereal crops, including wheat, barley, and rice [39]. PHS is a serious problem that causes yield and quality reduction in rice because it alters the physicochemical properties of starch. This results in reduced amylose content and irregular starch granules, leading to a deterioration in appearance and processing quality [40]. PHS is a major problem across a wide range of rice-cultivating regions, including the Republic of Korea, China, Japan, Europe, and Australia, and results in an annual economic loss of USD one billion on a global scale [41]. Achieving PHS resistance is therefore one of the most important targets for breeding programs in rice. To date, 185 QTLs linked to PHS and related traits, such as seed dormancy and low-temperature germination, have been identified [41]. To test the applicability of the target capture sequencing SNP genotyping platform to QTL analysis, we performed an analysis of PHS resistance with an F_8_ RIL population derived from a cross between two Korean temperate *japonica* rice varieties, Odae and Joun. Joun is resistant to PHS, whereas Odae is moderately resistant to PHS. We constructed a genetic map comprising 475 SNP markers (Figure 4) and found two QTLs, *qPHS4* and *qPHS11*, associated with PHS resistance. *qPHS4* was located in an interval between 29.751 and 32.749 Mbp on chromosome 4, whereas the region containing *qPHS11* was located between 14.468 and 15.592 Mbp on chromosome 11. A QTL associated with seed dormancy, *qSD4-2*, has previously been reported in the *qPHS4* region [42]. No QTL for PHS or seed dormancy has been detected previously in the *qPHS11* region, however, which indicates that *qPHS11* is a novel PHS QTL. As *qPHS11* is a major QTL with a high LOD score (14.74) and high PVE (28.66%), it may be productive to identify the gene(s) responsible for its effect using map-based cloning approaches. However, this QTL mapping was performed with PHS rate data measured in only 1 year. Therefore, PHS rates must be measured in another year or environment for complete QTL mapping.

## 5. Conclusions

We developed a target capture sequencing SNP genotyping platform containing 2341 SNP markers that were polymorphic across 50 rice varieties. The mean number of polymorphic markers for any combination of two rice varieties was high (854), which indicates that this platform has the potential to become a powerful tool for diverse types of genetic studies, including QTL and gene mapping analyses and the development of novel breeding programs. This platform was used to identify two QTLs for PHS resistance with an F_8_ RIL population derived from a cross between two Korean temperate *japonica* rice varieties, Odae and Joun.

## Figures and Tables

**Table 1:** Number of polymorphic markers for different variety pairs.

Group	Mean	Minimum	Maximum
All varieties	854	159	1368
Within *japonica*	623	159	916
Within *indica*	491	162	861
Between *japonica* and *indica*	1177	603	1368

**Table 2:** Identification of QTLs affecting PHS rate.

QTL name	Chromosome	Location (cM)	QTL Interval * (cM)	LOD	Additive Effect	PVE (%) **
*qPHS4*	4	126	121.5–126.5	4.06	−4.84	6.48
*qPHS11*	11	77	74.5–78.5	14.74	−10.13	28.66

* Interval at 95% probability. ** Phenotypic variation explained by QTL effects.

## Data Availability

Data is contained within the article or Supplementary Materials.

## Supplementary Materials

The following materials are available online at https://www.mdpi.com/article/10.3390/genes13050794/s1. Figure S1: Estimation of the most probable K value using LnP(D)-derived ΔK for K from 1 to 8. Table S1: List of varieties included in the diversity analysis using the target capture sequencing SNP genotyping platform. Table S2: List of all SNP markers included in the target capture sequencing SNP genotyping platform developed in this study. Table S3: Genotypes of rice varieties and PIC values of all markers used in the study. Table S4: Density across each chromosome of SNP markers present on the target capture sequencing SNP genotyping platform.

## References

[1] Wing R.A., Purugganan M.D., Zhang Q. (2018). The rice genome revolution: From an ancient grain to Green Super Rice. Nat. Rev. Genet..

[2] Chen H., Xie W., He H., Yu H., Chen W., Li J., Yu R., Yao Y., Zhang W., He Y. (2014). A High-Density SNP Genotyping Array for Rice Biology and Molecular Breeding. Mol. Plant.

[3] Zhao K., Wright M., Kimball J., Eizenga G., McClung A., Kovach M., Tyagi W., Ali L., Tung C.-W., Reynolds A. (2010). Genomic Diversity and Introgression in O. sativa Reveal the Impact of Domestication and Breeding on the Rice Genome. PLoS ONE.

[4] Thomson M.J., Zhao K., Wright M., McNally K.L., Rey J., Tung C.-W., Reynolds A., Scheffler B., Eizenga G., McClung A. (2011). High-throughput single nucleotide polymorphism genotyping for breeding applications in rice using the BeadXpress platform. Mol. Breed..

[5] Yamamoto T., Nagasaki H., Yonemaru J.-I., Ebana K., Nakajima M., Shibaya T., Yano M. (2010). Fine definition of the pedigree haplotypes of closely related rice cultivars by means of genome-wide discovery of single-nucleotide polymorphisms. BMC Genom..

[6] Yu H., Xie W., Li J., Zhou F., Zhang Q. (2014). A whole-genome SNP array (RICE 6 K) for genomic breeding in rice. Plant Biotechnol. J..

[7] Thomson M.J., Singh N., Dwiyanti M.S., Wang D.R., Wright M.H., Perez F.A., Declerck G., Chin J.H., Malitic-Layaoen G.A., Juanillas V.M. (2017). Large-scale deployment of a rice 6 K SNP array for genetics and breeding applications. Rice.

[8] Morales K.Y., Singh N., Perez F.A., Ignacio J.C., Thapa R., Arbelaez J.D., Tabien R.E., Famoso A., Wang D.R., Septiningsih E.M. (2020). An improved 7K SNP array, the C7AIR, provides a wealth of validated SNP markers for rice breeding and genetics studies. PLoS ONE.

[9] Zhao K., Tung C.-W., Eizenga G.C., Wright M., Ali M.L., Price A.H., Norton G., Islam M.R., Reynolds A.R., Mezey J.G. (2011). Genome-wide association mapping reveals a rich genetic architecture of complex traits in Oryza sativa. Nat. Commun..

[10] Singh N., Jayaswal P.K., Panda K., Mandal P., Kumar V., Singh B., Mishra S., Singh Y., Singh R., Rai V. (2015). Single-copy gene based 50 K SNP chip for genetic studies and molecular breeding in rice. Sci. Rep..

[11] McCouch S.R., Wright M., Tung C.-W., Maron L., McNally K., Fitzgerald M., Singh N., Declerck G., Agosto-Perez F., Korniliev P. (2016). Open access resources for genome-wide association mapping in rice. Nat. Commun..

[12] Thomson M.J. (2014). High-Throughput SNP Genotyping to Accelerate Crop Improvement. Plant Breed. Biotechnol..

[13] Arbelaez J.D., Dwiyanti M.S., Tandayu E., Llantada K., Jarana A., Ignacio J.C., Platten J.D., Cobb J., Rutkoski J.E., Thomson M.J. (2019). 1k-RiCA (1K-Rice Custom Amplicon) a novel genotyping amplicon-based SNP assay for genetics and breeding applications in rice. Rice.

[14] Guo Z., Wang H., Tao J., Ren Y., Xu C., Wu K., Zou C., Zhang J., Xu Y. (2019). Development of multiple SNP marker panels affordable to breeders through genotyping by target sequencing (GBTS) in maize. Mol. Breed..

[15] Guo Z., Yang Q., Huang F., Zheng H., Sang Z., Xu Y., Zhang C., Wu K., Tao J., Prasanna B.M. (2021). Development of high-resolution multiple-SNP arrays for genetic analyses and molecular breeding through genotyping by target sequencing and liquid chip. Plant Commun..

[16] Wang C., Liu R., Liu Y., Hou W., Wang X., Miao Y., He Y., Ma Y., Li G., Wang D. (2021). Development and application of the Faba_bean_130K targeted next-generation sequencing SNP genotyping platform based on transcriptome sequencing. Theor. Appl. Genet..

[17] Pootakham W., Shearman J.R., Ruang-Areerate P., Sonthirod C., Sangsrakru D., Jomchai N., Yoocha T., Triwitayakorn K., Tragoonrung S., Tangphatsornruang S. (2014). Large-Scale SNP Discovery through RNA Sequencing and SNP Genotyping by Targeted Enrichment Sequencing in Cassava (*Manihot esculenta* Crantz). PLoS ONE.

[18] Yang G., Chen S., Chen L., Sun K., Huang C., Zhou D., Huang Y., Wang J., Liu Y., Wang H. (2019). Development of a core SNP arrays based on the KASP method for molecular breeding of rice. Rice.

[19] Cheon K.-S., Baek J., Cho Y.-I., Jeong Y.-M., Lee Y.-Y., Oh J., Won Y.J., Kang D.-Y., Oh H., Kim S.L. (2018). Single Nucleotide Polymorphism (SNP) Discovery and Kompetitive Allele-Specific PCR (KASP) Marker Development with Korean Japonica Rice Varieties. Plant Breed. Biotechnol..

[20] Cheon K.-S., Jeong Y.-M., Lee Y.-Y., Oh J., Kang D.-Y., Oh H., Kim S.L., Kim N., Lee E., Baek J. (2019). Kompetitive Allele-Specific PCR Marker Development and Quantitative Trait Locus Mapping for Bakanae Disease Resistance in Korean Japonica Rice Varieties. Plant Breed. Biotechnol..

[21] Cheon K.-S., Jeong Y.-M., Oh H., Oh J., Kang D.-Y., Kim N., Lee E., Baek J., Kim S.L., Choi I. (2020). Development of 454 New Kompetitive Allele-Specific PCR (KASP) Markers for Temperate japonica Rice Varieties. Plants.

[22] Kang D.-Y., Cheon K.-S., Oh J., Oh H., Kim S.L., Kim N., Lee E., Choi I., Baek J., Kim K.-H. (2019). Rice Genome Resequencing Reveals a Major Quantitative Trait Locus for Resistance to Bakanae Disease Caused by Fusarium fujikuroi. Int. J. Mol. Sci..

[23] Cheon K.-S., Won Y.J., Jeong Y.-M., Lee Y.-Y., Kang D.-Y., Oh J., Oh H., Kim S.L., Kim N., Lee E. (2020). QTL mapping for pre-harvest sprouting resistance in japonica rice varieties utilizing genome re-sequencing. Mol. Genet. Genom..

[24] Shin Y., Won Y.J., Lee C., Cheon K.-S., Oh H., Lee G.-S., Baek J., Yoon I.S., Kim S.L., Cha Y.-S. (2022). Identification of Grain Size-Related QTLs in Korean *japonica* Rice Using Genome Resequencing and High-Throughput Image Analysis. Agriculture.

[25] Lee C.-M., Suh J.-P., Park H.-S., Baek M.-K., Jeong O.-Y., Yun S.-J., Cho Y.-C., Kim S.-M. (2021). Identification of QTL Combinations that Cause Spikelet Sterility in Rice Derived from Interspecific Crosses. Rice.

[26] Kang J.-W., Shin D., Cho J.-H., Lee J.-Y., Kwon Y., Park D.-S., Ko J.-M., Lee J.-H. (2019). Accelerated development of rice stripe virus-resistant, near-isogenic rice lines through marker-assisted backcrossing. PLoS ONE.

[27] Kim M.-S., Yang J.-Y., Yu J.-K., Lee Y., Park Y.-J., Kang K.-K., Cho Y.-G. (2021). Breeding of High Cooking and Eating Quality in Rice by Marker-Assisted Backcrossing (MABc) Using KASP Markers. Plants.

[28] Kim M.-S., Yu J.-K., Ko S.-R., Kim K.-J., Ji H., Kang K.-K., Cho Y.-G. (2022). Marker-Assisted Backcrossing (MABc) to Improve Eating Quality with Thin Seed Coat and Aleurone Layer of Non-Glutinous Japonica Variety in Rice. Genes.

[29] Sasaki A., Ashikari M., Ueguchi-Tanaka M., Itoh H., Nishimura A., Swapan D., Ishiyama K., Saito T., Kobayashi M., Khush G.S. (2002). Green revolution: A mutant gibberellin-synthesis gene in rice. Nature.

[30] Serrote C.M.L., Reiniger L.R.S., Silva K.B., Rabaiolli S.M.D.S., Stefanel C.M. (2020). Determining the Polymorphism Information Content of a molecular marker. Gene.

[31] Falush D., Stephens M., Pritchard J.K. (2003). Inference of Population Structure Using Multilocus Genotype Data: Linked Loci and Correlated Allele Frequencies. Genetics.

[32] Pritchard J.K., Stephens M., Donnelly P. (2000). Inference of population structure using multilocus genotype data. Genetics.

[33] Earl D.A., vonHoldt B.M. (2012). STRUCTURE HARVESTER: A website and program for visualizing STRUCTURE output and implementing the Evanno method. Conserv. Genet. Resour..

[34] Evanno G., Regnaut S., Goudet J. (2005). Detecting the number of clusters of individuals using the software structure: A simulation study. Mol. Ecol..

[35] Kumar S., Stecher G., Li M., Knyaz C., Tamura K. (2018). MEGA X: Molecular Evolutionary Genetics Analysis across Computing Platforms. Mol. Biol. Evol..

[36] Saitou N., Nei M. (1987). The neighbor-joining method: A new method for reconstructing phylogenetic trees. Mol. Biol. Evol..

[37] Tamura K., Nei M., Kumar S. (2004). Prospects for inferring very large phylogenies by using the neighbor-joining method. Proc. Natl. Acad. Sci. USA.

[38] Wang J., Li H., Zhang L., Meng L. (2016). Manual of QTL IciMapping.

[39] Lee J.-S., Chebotarov D., McNally K.L., Pede V., Setiyono T.D., Raquid R., Hyun W.-J., Jeung J.-U., Kohli A., Mo Y. (2021). Novel Sources of Pre-Harvest Sprouting Resistance for Japonica Rice Improvement. Plants.

[40] Zhu D., Qian Z., Wei H., Guo B., Xu K., Dai Q., Zhang H., Huo Z. (2019). The effects of field pre-harvest sprouting on the morphological structure and physicochemical properties of rice (*Oryza sativa* L.) starch. Food Chem..

[41] Sohn S.-I., Pandian S., Kumar T.S., Zoclanclounon Y.A.B., Muthuramalingam P., Shilpha J., Satish L., Ramesh M. (2021). Seed Dormancy and Pre-Harvest Sprouting in Rice—An Updated Overview. Int. J. Mol. Sci..

[42] Zhou Y., Xie Y., Cai J., Liu C., Zhu H., Jiang R., Zhong Y., Zhang G., Tan B., Liu G. (2017). Substitution mapping of QTLs controlling seed dormancy using single segment substitution lines derived from multiple cultivated rice donors in seven cropping seasons. Theor. Appl. Genet..

