# Supplementary figures and images for: Development and Application of a Target Capture Sequencing SNP-Genotyping Platform in Rice
Source: Genes (Basel). 2022 Apr 28;13(5):794. doi: 10.3390/genes13050794 (PMC9141132; doi:10.3390/genes13050794)

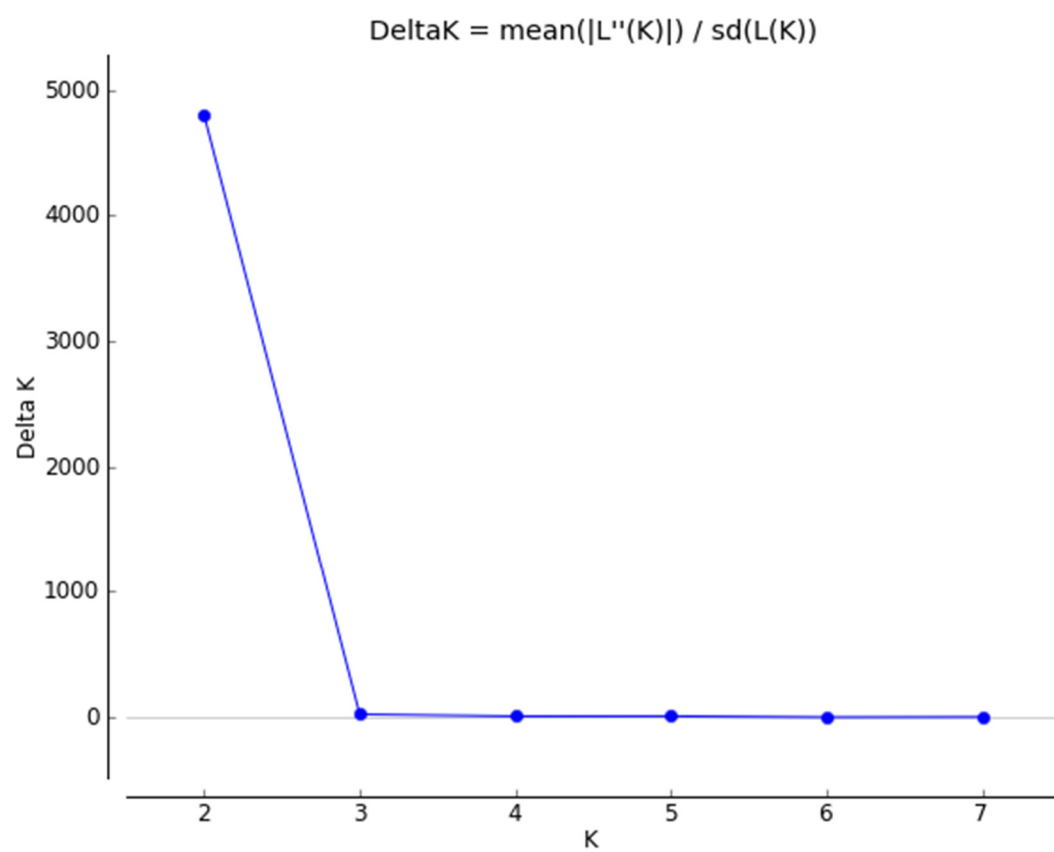

Figure S1. Estimation of the most probable  $K$  value using  $\text{LnP}(D)$  derived  $\Delta K$  for  $K$  from 1 to 8.

Supplement: Supplementary file 1 [file genes-13-00794-s001.zip › Figure_S1.pdf]
